# Spatial representations of numbers and letters in children

**DOI:** 10.3389/fpsyg.2013.00544

**Published:** 2013-08-21

**Authors:** Jan Lonnemann, Janosch Linkersdörfer, Telse Nagler, Marcus Hasselhorn, Sven Lindberg

**Affiliations:** ^1^Department of Education and Human Development, German Institute for International Educational Research (DIPF)Frankfurt am Main, Germany; ^2^Center for Individual Development and Adaptive Education of Children at Risk (IDeA)Frankfurt am Main, Germany; ^3^Department of Educational Psychology, Institute for Psychology, Goethe-UniversityFrankfurt am Main, Germany

**Keywords:** line bisection, mental number line, spatial representations, ordinal representations, cardinal representations

## Abstract

Different lines of evidence suggest that children's mental representations of numbers are spatially organized in form of a mental number line. It is, however, still unclear whether a spatial organization is specific for the numerical domain or also applies to other ordinal sequences in children. In the present study, children (*n* = 129) aged 8–9 years were asked to indicate the midpoint of lines flanked by task-irrelevant digits or letters. We found that the localization of the midpoint was systematically biased toward the larger digit. A similar, but less pronounced, effect was detected for letters with spatial biases toward the letter succeeding in the alphabet. Instead of assuming domain-specific forms of spatial representations, we suggest that ordinal information expressing relations between different items of a sequence might be spatially coded in children, whereby numbers seem to convey this kind of information in the most salient way.

## Introduction

Evidence for an association between number and space processing comes from behavioral experiments, patient examinations, and functional brain imaging studies (see Hubbard et al., 2005 for an overview). First indications of numerical-spatial interactions in children were presented by Berch et al. (1999), who demonstrated that typically developing children at the age of 9 years exhibited the so-called SNARC effect (Spatial Numerical Association of Response Codes; Dehaene et al., 1993). This effect reflects the observation that people respond faster with the left-hand side to smaller numbers than to larger numbers and vice versa for the right-hand side when being asked to compare numbers with respect to their magnitude or to classify numbers as even or odd (see Gevers and Lammertyn, 2005; Hubbard et al., 2005 for overviews). Later on, it could be demonstrated that even younger children at the age of 7 years exhibited the SNARC effect (van Galen and Reitsma, 2008). Further indications of interactions between number and space processing in children were detected in a so-called numerical landmark test (Lonnemann et al., 2008). The authors asked children aged 8–9 years to decide which of the two numerical distances in a visually presented number triplet was numerically larger. Numerical and spatial distances were manipulated independently, resulting in congruent (e.g., 57_64___92) and incongruent (e.g., 57___64_92) conditions. The spatial distances between the numbers clearly affected the comparison of numerical distances: Reaction times were faster and error rates smaller for congruent than for incongruent trials.

These findings are predominantly explained by a left-to-right oriented “mental number line” (Restle, 1970), comprising semantic (i.e., cardinal) representations of numbers (Dehaene et al., 1993). The SNARC effect, for instance, has been explained in terms of an irrepressible correspondence between the position of response modalities and the position of a respective number on the mental number line (Dehaene et al., 1993). This approach has, however, been put into question. Instead of a direct mapping of numerical magnitude representations (in form of a mental number line) to response locations, an alternative model has been proposed that entails an intermediate step between number magnitude and response representations, in which numbers are categorized as either small or large (Gevers et al., 2006; see also Chen and Verguts, 2010). Indeed, the above mentioned findings suggesting numerical-spatial interactions in children were based on bimanual left-right response settings and may have merely resulted from an association between verbal concepts such as “small” and “left” as well as “large” and “right” without any visuo-spatial coding (Proctor and Cho, 2006; Gevers et al., 2010; Imbo et al., 2012).

The assumption of a spatial layout of mental number representations in children is, however, also supported by findings which are not based on bimanual left-right response settings. For instance, van Galen and Reitsma (2008) presented (irrelevant) single digit numbers at fixation, followed by a target in either the left visual field (LVF) or the right visual field (RVF) which had to be detected by responding with the preferred hand. The presentation of relatively small digits facilitated the response to targets in the LVF, whereas relatively large numbers gave rise to faster detection of targets in the RVF. Although a SNARC effect was found in 7-, 8-, and 9-year-old children, these attentional shifts induced by numbers could only be detected in 9-year-olds. The authors thus assumed that younger children cannot automatically access numerical magnitude information when perceiving Arabic numerals. A study by de Hevia and Spelke (2009), however, indicated that such an automatic activation of numerical magnitude information, which might be spatially coded, could already be detected in younger children. The authors employed a line bisection task, in which adults and 7 year-old children were asked to indicate the midpoint of horizontal lines flanked by two different digits or by two different arrays of dots. It could be demonstrated that the localization of the midpoint was systematically biased toward the larger digit or magnitude of dots. Based on non-symbolic numerical displays, this effect was also observed in children prior to the onset of formal schooling. The authors concluded that numerical and spatial representations are intrinsically linked. A recent study by Gebuis and Gevers (2011), however, challenged this interpretation by showing that the bisection bias could be attributed to a larger area subtended by the arrays of dots with larger magnitudes. Therefore, a non-numerical explanation seems more apt to explain these results. Differences in non-numerical perceptual cues, however, can hardly explain the abovementioned effect for symbolic flankers detected in adults and in some 7-year-old children (12 out of 20 children showed an effect, see de Hevia and Spelke, 2009). These effects might be taken as first evidence for the emergence of a mental number line representation in children at the age of 7 years.

Using the line bisection task in adults, however, de Hevia et al. (2006) demonstrated that the spatial bias toward the larger magnitude is not modulated by the numerical distance between the digit flankers. In light of this finding, the authors questioned the adequacy of an interpretation in terms of a mental number line involving cardinal representations of numbers. Instead, they assumed that spatial bias arises from a spatial organization of categorical or relative magnitude information (see Nathan et al., 2009 for a similar view). Within this perspective, any kind of ordered information may be spatially organized. For adults, there is evidence that not only numbers (Fischer et al., 2003) but also non-numerical ordinal sequences such as letters, days, and months can induce spatial shifts of attention (Dodd et al., 2008). In contrast to numerical stimuli, however, non-numerical stimuli need to be processed in an order-relevant fashion (i.e., determining whether a particular item comes before or after an item in the middle of the respective ordinal sequence) to elicit these attentional shifts. According to the authors it is possible that numbers convey ordinal information in a more salient manner than the other sequence types.

Children's conception of how numerical magnitudes map onto a spatial scale, indexed by how numbers are placed on “number lines” (e.g., horizontal lines with 0 at one end and 100 at the other), seems to change during the first years of school, shifting from a logarithmic to a linear mapping (Siegler and Opfer, 2003; Siegler and Booth, 2004). Recently, a similar developmental trend could be demonstrated for non-numerical ordinal sequences like letters and months, suggesting that numbers and non-numerical sequences also share common representational mechanisms in children (Berteletti et al., 2012). Asking children to place numbers, letters, or months on a line, however, enforces spatial representations of the respective information. It is therefore still unclear whether children spontaneously represent numbers as well as non-numerical sequences in a spatial form. The present study was designed to address this issue. We employed the line bisection task used by de Hevia and Spelke (2009). By solely asking children to indicate the midpoint of a line flanked by task-irrelevant symbols, this task allows to capture spontaneous spatial biases. Since de Hevia and Spelke (2009) detected flanker effects of digits only in some 7-year-old children, we decided to examine children aged 8–9 years. In addition to lines flanked by two single digits, we decided to use lines flanked by two single letters, as both types of stimulus are perceptually comparable, overlearned sequences. Detection of similar effects for digits and letters would suggest that a spatial organization of children's mental representations is not specific for the numerical domain.

## Materials and methods

### Participants

Participants were 129 (65 females) third graders (mean age: 9 years, 1 month; *SD* = 6 months) recruited from 8 primary schools in and around Wiesbaden (Germany). Written and informed consent was obtained from all parents involved.

### Materials

**Figure 1:**
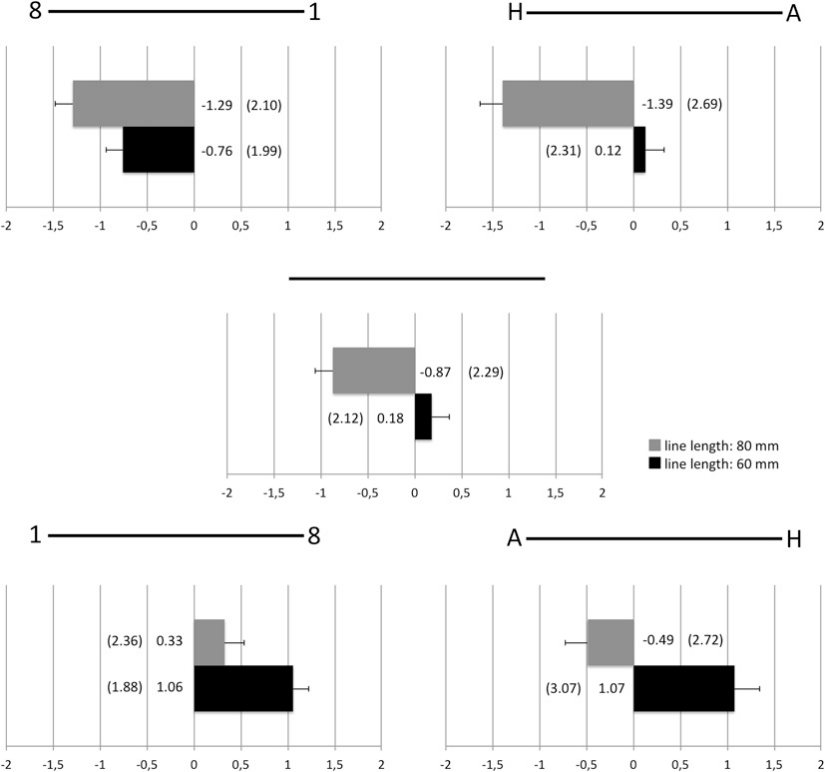
**Mean bisection deviations (mm; standard deviations in parentheses) for lines flanked by digits, lines flanked by letters, and lines without flankers separately for the two different line lengths**.

The stimulus material was adopted from de Hevia and Spelke (2009). Stimuli were horizontal black lines, 1 mm in width and either 60 or 80 mm in length, presented in the center of a horizontally oriented sheet (210 × 297 mm). The numerals “1” and “8” or the letters “A” and “H” appeared 1 mm to the left and right of the line, each about 5 mm wide and about 7 mm high (see Figure 1). de Hevia and Spelke (2009) used “2” and “9” as flankers, but as the corresponding letters (i.e., the second and the ninth letter of the alphabet) would have included an “I” which resembles “1,” we decided to use “1” and “8” as well as “A” and “H.” To determine unbiased line bisection performance of the children and to delineate possible flanker effects from these scores, a control condition without flankers was added.

### Design

The experiment consisted of two 32-trial blocks (2 flanker sides × 2 line lengths × 8 repetitions), one block with digits and the second one with letters as flankers. Additionally, a 16-trial control block (2 line lengths × 8 repetitions) was conducted, in which no flankers were shown. These three different blocks were presented in alternation and the order of blocks was counterbalanced across subjects. The order of trials within each block was pseudo-randomized so that neither the flanker side nor the line length was identical on more than three consecutive trials.

### Procedure

Stimuli were presented one at a time, aligned with reference to the mid-saggital plane of the body. Children were instructed to mark the center of each line accurately and rapidly using a pencil with their preferred hand. The flanking numbers/letters were not mentioned. Data collection was conducted in groups of about 20 children.

### Analyses

Bisection marks were measured to the nearest millimeter using a ruler. Deviations from the objective center of the line to the left were expressed as negative and deviations to the right as positive values. Mean values for each child were submitted to a 2 by 2 by 2 repeated-measures analysis of variance (ANOVA) with the factors *flanker content* (digits vs. letters), *flanker side* (“8” or “H” on left vs. right side), and *line length* (60 vs. 80 mm). Lines without flankers were analyzed separately by an ANOVA with the factor *line length* (60 vs. 80 mm). Follow-up paired-sample *t*-tests were used for further investigations.

## Results

Analyzing mean scores for lines with flankers revealed significant main effects for flanker side [*F*_(1, 128)_ = 198.77, *p* < 0.001, partial eta-squared = 0.61] and for line length [*F*_(1, 128)_ = 25.67, *p* < 0.001, partial eta-squared = 0.17] as well as significant interactions between the factors flanker content and flanker side [*F*_(1, 128)_ = 29.40, *p* < 0.001, partial eta-squared = 0.19] and between flanker content and line length [*F*_(1, 128)_ = 16.26, *p* < 0.001, partial eta-squared = 0.11]. No other effects reached significance [flanker content: *F*_(1, 128)_ = 0.00, *p* = 0.960; flanker side × line length: *F*_(1, 128)_ = 0.63, *p* = 0.428; flanker content × flanker side × line length: *F*_(1, 128)_ = 0.18, *p* = 0.668].

The main effect for flanker side was characterized by rightward deviations (0.49 mm) for lines flanked by “8” or “H” on the right hand side and leftward deviations (−0.83 mm) for lines flanked by “8” or “H” on the left hand side. Follow-up *t*-tests revealed that effects of flanker side could be detected for digits [“8” on left side: −1.03 mm, “8” on right side: 0.70 mm, *t*_(128)_ = 18.37, *p* < 0.001 (two-sided)] as well as for letters [“H” on left side: −0.64 mm, “H” on right side: 0.30 mm, *t*_(128)_ = 6.63, *p* < 0.001 (two-sided)]. Compared with lines flanked by letters, however, lines flanked by digits yielded more positive deviations in case of “8” (“H” for lines flanked by letters) on the right hand side [digits: 0.70 mm, letters: 0.30 mm, *t*_(128)_ = 2.30, *p* < 0.05 (two-sided)] and more negative deviations in case of “8” (“H” for lines flanked by letters) on the left hand side [digits: −1.03 mm, letters: −0.64 mm, *t*_(128)_ = 3.27, *p* = 0.001 (two-sided)]. As a result, determining the midpoint of horizontal lines was systematically biased not only by digits but also by letters. The interaction between flanker content and flanker side, however, revealed that digits elicited a stronger spatial bias.

The main effect for line length showed rightward deviations (0.38 mm) for short lines and leftward deviations (−0.71 mm) for longer lines. Follow-up *t*-tests revealed effects of line length for lines flanked by digits [short lines: 0.15 mm, long lines: −0.48 mm, *t*_(128)_ = 3.48, *p* = 0.001 (two-sided)] as well as for lines flanked by letters [short lines: 0.60 mm, long lines: −0.94 mm, *t*_(128)_ = 5.30, *p* < 0.001 (two-sided)]. In comparison with lines flanked by letters, however, lines with digit flankers yielded less positive deviations in case of short lines [digits: 0.15 mm, letters: 0.60 mm, *t*_(128)_ = 2.26, *p* < 0.05 (two-sided)] and less negative deviations in case of long lines [digits: −0.48 mm, letters: −0.94, *t*_(128)_ = 3.22, *p* < 0.01 (two-sided)]. The interaction between flanker content and line length could therefore be attributed to stronger effects of line length for letters.

**Figure 2:**
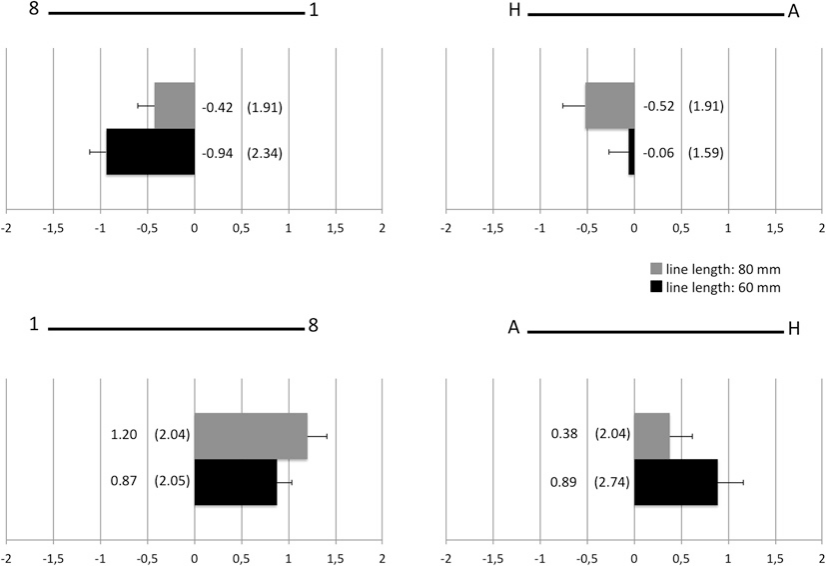
**Difference values (mm) between mean scores for lines with and without flankers (standard deviations in parentheses) for lines flanked by digits and lines flanked by letters separately for the two different line lengths**.

As can be seen in Figure 1, digits induced leftward deviations if the larger digit was displayed on the left side and rightward deviations if the larger digit was displayed on the right side of the line. On the other hand, letters induced rightward deviations for short lines and leftward deviations for longer lines. Results of the line bisection task without flankers, however, give meaning to these findings. Similarly to the findings for lines with flankers, mean scores for lines without flankers revealed an effect of line length with leftward deviations for longer lines and rightward deviations for shorter lines [short lines: 0.18 mm, long lines: −0.87 mm, *t*_(128)_ = 4.03, *p* < 0.001 (two-sided)]. Controlling for the subjectively perceived centers of unflanked lines by computing difference values between mean scores for lines with and without flankers (e.g., the mean score for 60 mm long lines without flankers was subtracted from the mean score for 60 mm long lines with “1” to the left and “8” to the right of the line) revealed that letters similar to digits induced leftward deviations in case of the “H” on the left and rightward deviations in case of the “H” on the right hand side (see Figure 2).

## Discussion

The present study was designed to answer the question whether in children a spatial organization of mental representations is specific for the numerical domain or also applies to other non-numerical ordinal sequences. We asked children to indicate the midpoint of lines flanked by task-irrelevant digits or letters. In concert with earlier findings (e.g., de Hevia and Spelke, 2009), it could be demonstrated that the localization of the midpoint was systematically biased toward the larger digit. According to de Hevia and Spelke (2009) this finding represents evidence for a spontaneous spatial representation of numerical magnitude (i.e., cardinal) information in form of a mental number line. The present study, however, revealed a similar effect for letters with bias toward the letter succeeding in the alphabet, indicating that a spontaneous spatial coding of information is not specific for the numerical domain. Instead of assuming domain-specific forms of spatial representations like a mental number line or an “alphabet line” for letters (Berteletti et al., 2012), the spatial bias for digits and letters might be explained by the notion that spatial coding comes into play whenever relations are established (Nathan et al., 2009). Indeed, each line in the bisection task was flanked by two different digits/letters, thereby building up a relation. Furthermore, it has already been shown in adults that strings of identical digits, involving small or large magnitudes, are bisected similarly, demonstrating that the absolute magnitude of numbers is not sufficient to modulate performance in the bisection task (de Hevia et al., 2006). This line of reasoning on its own, however, can hardly be applied to explain that numbers automatically elicit spatial shifts of attention in adults (Fischer et al., 2003) as well as in children (van Galen and Reitsma, 2008) but non-numerical stimuli such as letters need to be processed in an order-relevant fashion to induce spatial attention shifts in adults (Dodd et al., 2008). Accordingly, domain-specific processing mechanisms seem to exist for numbers. Indeed, our findings also indicate modality-specific differences, with digits eliciting stronger spatial biases than letters. It might be possible that cardinal information, which is only provided by digits, provoked these modality-specific differences by strengthening flanker effects for digits. However, as varying the numerical distance between the two flanking digits does not modulate spatial bias in the line bisection task (see de Hevia et al., 2006), cardinal information does not seem to play a role, at least in adults. Instead, the modality-specific differences might be explained by the notion raised by Dodd et al. (2008) that numbers convey ordinal information in a more salient manner than other sequence types. As ordinality always expresses a relation between different items of a sequence, this idea might be conjoined with the abovementioned suggestion that spatial coding comes into play whenever relations are established (Nathan et al., 2009): Ordinal information expressing relations between different items of a sequence might be spatially coded in children, numbers conveying this kind of information in the most salient way.

Even though the stimulus material of the present study was adapted to that used by de Hevia and Spelke (2009), we found an effect of line length with rightward deviations for short lines and leftward deviations for longer lines, which was not reported by de Hevia and Spelke (2009). However, a leftward bisection error for long lines is well-known as “pseudoneglect” (see Jewell and McCourt, 2000, for an overview) and the “cross-over” to a reversed bias for short lines has been reported in studies confronting patients with hemi-neglect (e.g., Halligan and Marshall, 1988) as well as neurologically normal participants (e.g., McCourt and Jewell, 1999) with the line bisection task. The absence of these effects in all of the experiments reported by de Hevia and Spelke (2009) might be ascribed to a lack of power due to small sample sizes (no more than 25 participants per experiment). Surprisingly, effects of line length detected in the present study were differently pronounced for the different flanker contents. Effects of line length were, however, not central to the present study and have to be interpreted with caution, especially because only two different line lengths were used.

A limitation of the present study resides in the use of only one set of digits and one set of letters. The reason for this restriction was the attempt to adapt the stimulus material to the one used by de Hevia and Spelke (2009), in order to answer the question whether a spatial organization of mental representations is specific for the numerical domain. While it could be demonstrated that not only numbers but also letters seem to be spatially coded in children, the stimulus set of the present study did not allow for ruling out that cardinal information provoked modality-specific differences by strengthening flanker effects for digits. We argue that cardinal information does not seem to play a role because varying the numerical distance between the two flanking digits does not modulate spatial biases in the line bisection task in adults (see de Hevia et al., 2006). Whether this also applies to children, however, has yet to be clarified.

In conclusion, results from our study revealed spontaneous spatial mappings of numbers and letters in children aged 8–9 years. We suggest that ordinal information expressing relations between different items of a sequence might be spatially coded in children, whereby numbers seem to convey this kind of information in the most salient way. As other recent findings highlight the role of ordinal information processing in numerical cognition (Lyons and Beilock, 2011; Rubinsten and Sury, 2011), more careful examination of this topic may provide important information regarding the development of numerical competencies.

### Conflict of interest statement

The authors declare that the research was conducted in the absence of any commercial or financial relationships that could be construed as a potential conflict of interest.
